# Curcumin mediates polyamine metabolism and sensitizes gastrointestinal cancer cells to antitumor polyamine-targeted therapies

**DOI:** 10.1371/journal.pone.0202677

**Published:** 2018-08-23

**Authors:** Tracy Murray-Stewart, Matthew Dunworth, Yuan Lui, Francis M. Giardiello, Patrick M. Woster, Robert A. Casero

**Affiliations:** 1 Sidney Kimmel Comprehensive Cancer Center, Johns Hopkins University School of Medicine, Baltimore, MD, United States of America; 2 Department of Medicine, Johns Hopkins University School of Medicine, Baltimore, MD, United States of America; 3 Department of Pathology, Johns Hopkins University School of Medicine, Baltimore, MD, United States of America; 4 Department of Drug Discovery and Biomedical Sciences, College of Pharmacy, Medical University of South Carolina, Charleston, SC, United States of America; ENEA Centro Ricerche Casaccia, ITALY

## Abstract

Curcumin, a natural polyphenol that contributes to the flavor and yellow pigment of the spice turmeric, is known for its antioxidant, anti-inflammatory, and anticarcinogenic properties. Capable of affecting the initiation, promotion, and progression of carcinogenesis through multiple mechanisms, curcumin has potential utility for both chemoprevention and chemotherapy. Previous studies demonstrated that curcumin can inhibit ornithine decarboxylase (ODC) activity in human leukemia and breast cancer cells, and pretreatment with dietary curcumin blocks carcinogen-induced ODC activity in rodent models of skin, colon, and renal cancer. The current study investigated the regulation of polyamine metabolism in human gastric and colon carcinoma cell lines in response to curcumin. Curcumin treatment significantly induced spermine oxidase (SMOX) mRNA and activity, which results in the generation of hydrogen peroxide, a source of ROS. Simultaneously, curcumin down regulated spermidine/spermine *N*^1^-acetyltransferase (SSAT) activity and the biosynthetic enzymes ODC and S-adenosylmethionine decarboxylase (SAMDC), thereby diminishing intracellular polyamine pools. Combination treatments using curcumin with the ODC inhibitor 2-difluoromethylornithine (DFMO), an agent currently in clinical chemoprevention trials, significantly enhanced inhibition of ODC activity and decreased growth of GI cancer cell lines beyond that observed with either agent alone. Similarly, combining curcumin with the polyamine analogue bis(ethyl)norspermine enhanced growth inhibition that was accompanied by enhanced accumulation of the analogue and decreased intracellular polyamine levels beyond those observed with either agent alone. Importantly, cotreatment with curcumin permitted the lowering of the effective dose of ODC inhibitor or polyamine analogue. These studies provide insight into the polyamine-related mechanisms involved in the cancer cell response to curcumin and its potential as a chemopreventive or chemotherapeutic agent in the GI tract.

## Introduction

**Fig 1:**
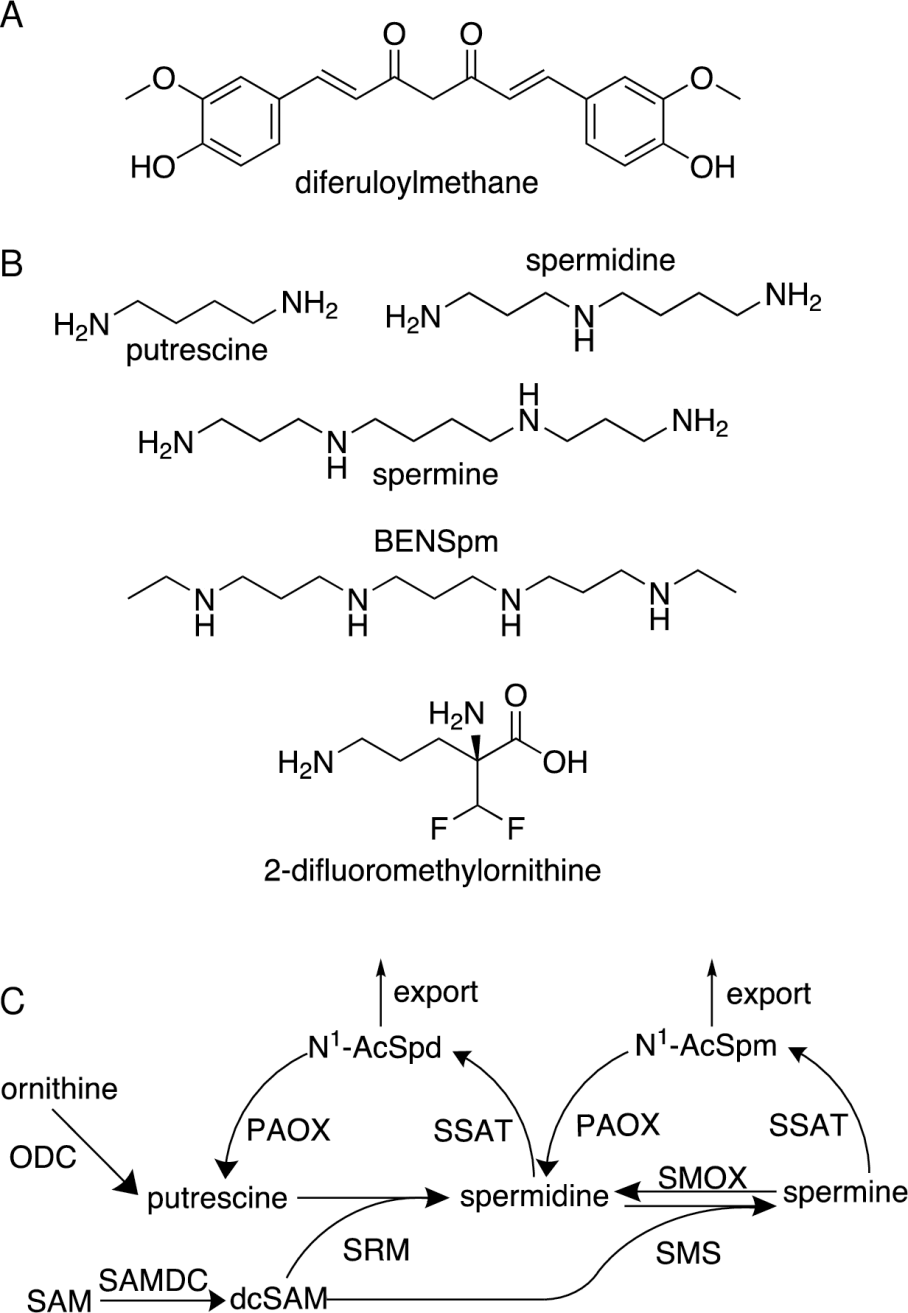
Chemical structures and the polyamine pathway. (A) Chemical structure of curcumin (diferuloylmethane). (B) Chemical structures of the natural polyamines spermine, spermidine, and putrescine, the polyamine analogue bis(ethyl)norspermine (BENSpm), and the ODC inhibitor difluoromethylornithine (DFMO). (C) The mammalian polyamine pathway: ornithine decarboxylase (ODC) converts ornithine into putrescine, which acquires sequential aminopropyl groups to form spermidine followed by spermine. The aminopropyl group is derived from decarboxylated S-adenosylmethionine (dcSAM), and its addition onto putrescine or spermidine is mediated by spermidine and spermine synthases (SRM and SMS, respectively). Two routes exist for spermine catabolism: (1) it can be oxidized by spermine oxidase (SMOX) to produce spermidine, which is accompanied by H_2_O_2_ and aldehyde generation, or (2) it can undergo acetylation by spermidine/spermine N^1^-acetyltransferase (SSAT). Acetylated spermine is either exported from the cell or oxidized back to spermidine by peroxisomal N^1^-acetylpolyamine oxidase (PAOX). Spermidine also undergoes this 2-step acetylation/oxidation back to putrescine via an N^1^-acetylspermidine intermediate.

Curcumin, or diferuloylmethane (1,7-bis(4-hydroxy 3-methoxy phenyl)-1,6-heptadiene-3.5-dione) (Fig 1A) accounts for the characteristic yellow-orange color and flavor of the spice turmeric. Curcuminoids constitute approximately 5% of the crude extract of turmeric root (*Curcuma longa*), of which curcumin (diferuloylmethane) is the most abundant (60–70%), followed by desmethoxycurcumin (20–27%), and bisdesmethoxycurcumin (10–15%)[1]. As a naturally occurring bioactive polyphenol, curcumin has been used for centuries in traditional medicines for relief from a wide variety of ailments. In addition, it is commonly used in cooking as a spice and food coloring, in skin care products, and as a textile dye. Thus, there exists a long history of safe curcumin use in human populations.

The purported health-promoting properties of curcumin are diverse and include antioxidant, anti-inflammatory, antiproliferative, anti-angiogenic, and antibacterial activities. This multitude of curcumin effects can likely be attributed to its capacity for modulating certain key signaling pathways with roles in multiple pathologies, including cancer [2]. In addition to chemopreventive potential, curcumin has demonstrated anticancer effects on established tumor cells, which, in contrast to non-tumorigenic cells, often undergo apoptosis and cell death upon exposure to curcumin [3].

The polyamine metabolic pathway plays significant roles in all stages of cancer development. Cancer cells of all types display elevations in polyamine biosynthesis, resulting in intracellular concentrations of polyamines that exceed those of their normal counterparts [4–7]. This consistency across tumor types highlights the importance of polyamines in tumorigenesis and as a potential drug target. Mammals contain three main polyamines, spermine, spermidine, and putrescine (Fig 1B), which, due to their positive charge at physiological pH, readily interact with negatively charged macromolecules, including DNA, RNA, certain proteins, membranes, and ion channels. As a result, polyamines have the ability to affect multiple critical cell processes, including DNA replication, transcription, translation, autophagy, and apoptosis. Thus, their levels must be maintained via tightly controlled regulation.

The dysregulated polyamine metabolism of neoplastic cells involves up regulation of biosynthetic enzymes, down regulation of catabolic enzymes, and increased polyamine uptake from the extracellular environment (Fig 1C) [5, 8–10]. Critical to this loss of homeostasis is an increase in ornithine decarboxylase (ODC) activity, the initial rate-limiting biosynthetic step converting the amino acid ornithine into the diamine putrescine. Rapidly induced by oncogenic stimuli, chemical carcinogens, and tumor promoters, ODC activity is essential for malignant transformation and oncogenesis [9, 11], and the ability of a compound to block this ODC induction is considered an indicator of chemopreventive potential [12]. This is best exemplified by the irreversible ODC inhibitor 2-difluoromethylornithine (DFMO, Fig 1B), or eflornithine, which has shown particular promise in clinical trials involving patients predisposed to colorectal tumors [13]. Importantly, curcumin prevents carcinogen- or tumor promoter-induced ODC activity and tumor formation in rodent models of skin, renal, and colon carcinoma (reviewed in [14]). Curcumin also inhibits ODC activity, decreases polyamine levels, and induces apoptosis in multiple breast cancer cell lines [15, 16] and in a promyelocytic leukemia cell line [17].

Therefore, curcumin has demonstrated utility in targeting both the development of cancer and established tumors, and the polyamine metabolic pathway can be targeted to inhibit carcinogenesis in predisposed populations and as a chemotherapeutic target. As the potential for long-term exposure to dietary factors is greatest in the gastrointestinal (GI) tract, the current study comprehensively investigates the effects of curcumin on gastric and colon cancer cell lines with regard to polyamine metabolism including the catabolic pathway. We report for the first time that curcumin strongly up regulates spermine oxidase (SMOX) while down regulating SSAT and the biosynthetic pathway. The mechanistic importance of these changes is evaluated in the cytotoxic response to curcumin, and novel evidence is provided suggesting the clinical utility of curcumin in combination with existing modulators of the polyamine pathway in chemopreventive and chemotherapeutic strategies.

## Materials and methods

### Cell lines and culture conditions

AGS gastric adenocarcinoma cells (ATCC #CRL-1739, Manassas, VA) were maintained in DMEM/F-12 medium (Mediatech, Manassas, VA) containing 10% fetal calf serum, penicillin and streptomycin at 37°C, 5% CO_2_. HCT116 colon carcinoma cells (ATCC #CCL-247) were maintained in McCoy’s 5A medium supplemented with 10% fetal calf serum, penicillin and streptomycin. Pure curcumin provided by the National Cancer Institute was dissolved in sterile DMSO at a concentration of 1 mg/mL and stored at -20°C in single-use aliquots. The polyamine oxidase inhibitor MDL72527 [18] and the polyamine analogue bis(ethyl)norspermine (BENSpm) [19] were synthesized as previously reported. The ornithine decarboxylase inhibitor DFMO was from the Department of Drug Discovery and Biomedical Sciences, Medical University of South Carolina. Spermine, spermidine, and aminoguanidine were purchased from Sigma (St. Louis, MO).

### Cell proliferation assays

AGS or HCT116 cells were seeded in triplicate wells per condition of 96-well plates and allowed to attach overnight. Cells were then treated with 100 μL of fresh medium containing the appropriate concentrations of curcumin, DFMO, BENSpm, MDL72527, and/or 5 μM spermine or spermidine in the presence of 1 mM aminoguanidine to prevent extracellular oxidation by bovine serum oxidase. Following the indicated incubation times, cell viability was determined using the CellTiter 96 AQueous One Solution Cell Proliferation assay (Promega, Madison, WI). Wells without cells were used as blanks for each condition to account for possible variations in absorbance due to the presence of curcumin.

### RNA isolation and quantitative reverse-transcriptase PCR

Total RNA was isolated from treated cells using TRIzol reagent according to the manufacturer’s protocol (Invitrogen, Carlsbad, CA). One μg of RNA was used for cDNA synthesis using qScript cDNA SuperMix (Quanta Biosciences, Gaithersburg, MD), followed by SYBR green-mediated real-time PCR (Universal SYBR Green Supermix, BioRad, Hercules, CA) using custom primers specific for *SMOX*, *SAT1*, *AMD1*, and *ODC* (Integrated DNA Technologies, Coralville, IA). Primer pairs were previously optimized using annealing temperature gradients with melt curve analyses and visualization on 2% agarose gels. In each experiment, samples were performed in triplicate, normalized to *GAPDH* as an internal control, and fold change in expression relative to untreated cDNA was determined using the 2^-ΔΔCt^ algorithm. Thermocycling was performed on a Bio-Rad iQ2 real-time PCR detection system, with data collection facilitated by the iQ5 optical system software.

### Enzyme assays and intracellular polyamine pool determinations

Lysates from treated cells were used for ODC, SAMDC, and SSAT enzyme activity assays according to previously reported methods [20–22]. Acid-extracted lysate aliquots were labeled with dansyl chloride for fluorometric detection using HPLC as previously described [23]. All enzyme activities and polyamine concentrations were quantified relative to total cellular protein in the lysate, as determined by the method of Bradford [24].

### Western blot analyses

Following treatment, cells were lysed in 4% SDS containing protease inhibitors and passed through a homogenizer column (Zymo Research, Irvine, CA). Protein was quantified using the BioRad DC assay with interpolation on a bovine serum albumin standard curve. Reduced samples (30 μg/lane) were separated on 4–12% Bis-Tris BOLT gels (Invitrogen), followed by transfer onto Immun-Blot PVDF (BioRad) and blocking in Odyssey blocking buffer (LI-COR, Lincoln, NE) at room temperature for 1 hour. Membranes were incubated with primary antibodies targeting SMOX, SSAT, ODC, γH2AX (Abcam, Cambridge, MA), and ß-actin (Santa Cruz Biotechnology, Santa Cruz, CA) overnight at 4°C. Species-specific, fluorophore-conjugated secondary antibodies were used for visualization and quantitation of bands using an Odyssey infrared detection system and software (LI-COR).

### CRISPR-Cas9-mediated generation of SMOX-knockout cell lines

SMOX-knockout cell lines were generated using the CRISPR-Cas9 system. Briefly, single guide RNA (5’-ACCCTCTCAGTCGCGGCCTA-3’) targeting the *SMOX* gene was cloned into the lentiCRISPR plasmid and viral particles were packaged in HEK293T (ATCC #CRL-3216) cells according to a previously published protocol [25]. Lentiviral particles were then used to transduce AGS cells, and individual clones were selected for resistance to puromycin. Expanded colonies were screened for SMOX knockout by Western blotting.

### Statistical analyses

Statistically significant differences were determined as those with p-values less than 0.05, as determined by Student’s t-test using GraphPad software (La Jolla, CA). For combination studies, statistical significance (p < 0.05) was determined by one-way ANOVA with post-hoc analyses.

## Results

### Curcumin reduces polyamine biosynthesis and intracellular polyamine pools in AGS cells

**Fig 2:**
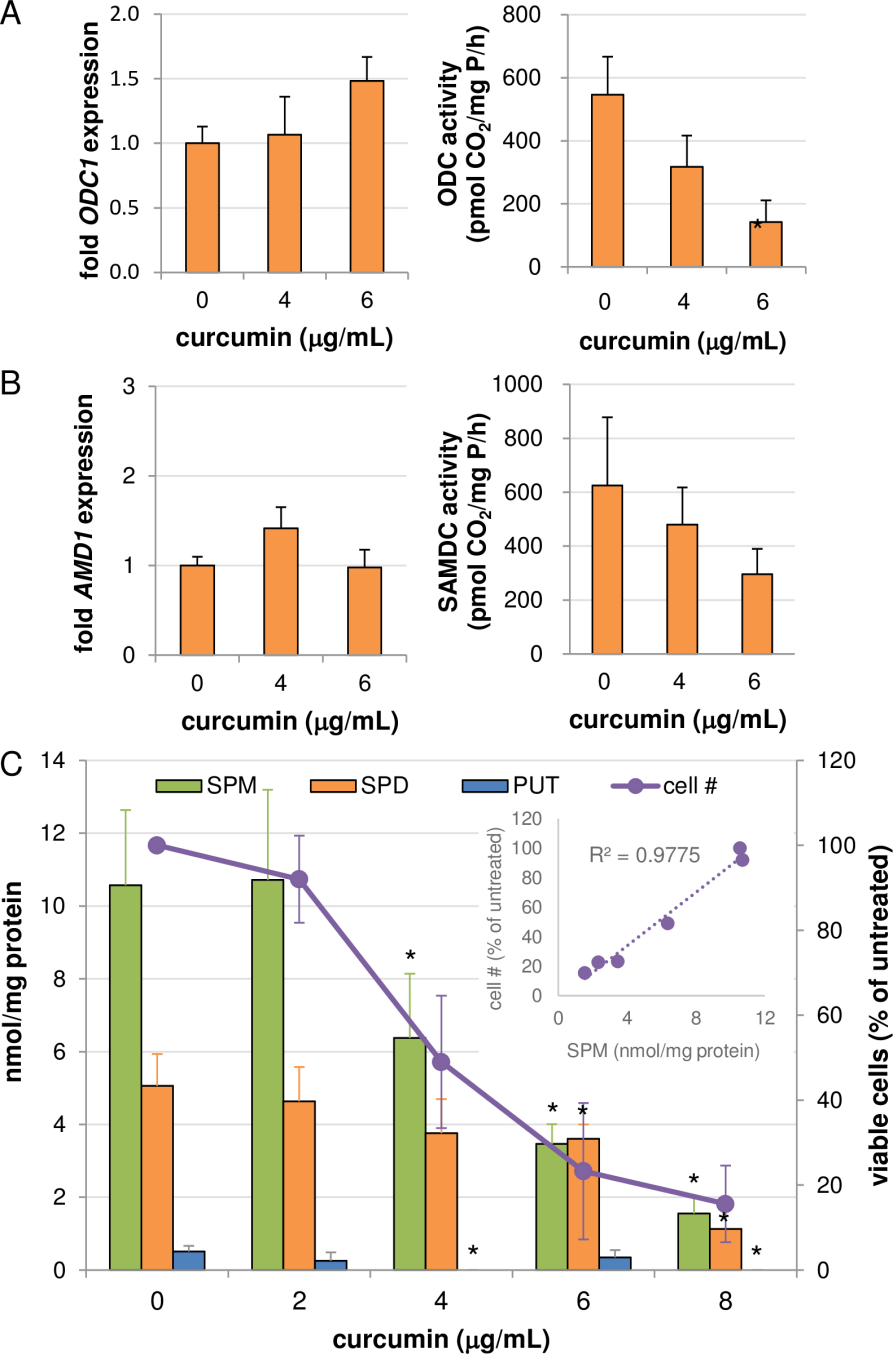
Effects of curcumin on polyamines and their biosynthetic enzymes in AGS cells. AGS gastric carcinoma cells were treated for 48 h with curcumin followed by analyses of mRNA expression and enzyme activity levels of the polyamine biosynthetic enzymes ODC (A) and SAMDC (encoded by the *AMD1* gene) (B). (C) Curcumin decreased intracellular polyamine pools and inhibited growth of AGS cells (SPM: spermine; SPD: spermidine; PUT: putrescine). (Inset) Linear regression of spermine concentration versus % of viable cells remaining relative to untreated cells in response to increasing doses of curcumin (*p < 0*.*001*). Graphs depict the means of data collected from at least 2 separate biological experiments, each measured in triplicate (duplicate for HPLC), with error bars indicating standard error of the mean. *p < 0.*05* relative to untreated.

As curcumin inhibits ODC activity in breast cancer and leukemia cell lines [15, 17], we first determined its effect on ODC mRNA and activity in cancer cell lines of gastrointestinal origin. Not surprisingly, treatment of AGS gastric cancer cells with increasing concentrations of pure curcumin for 48 hours resulted in a dose-dependent decrease in ODC activity, although this decrease was not associated with altered levels of ODC mRNA (Fig 2A). Similarly, curcumin treatment resulted in a non-statistically significant reduction in the enzyme activity of a second rate-limiting enzyme in polyamine biosynthesis, *S*-adenosylmethionine decarboxylase (SAMDC), without affecting its mRNA transcript level (Fig 2B). HPLC analyses revealed dose-dependent decreases in intracellular concentrations of each of the 3 natural polyamines (Fig 2C). Interestingly, the largest observed reduction was in the higher order polyamine spermine, and this reduction strongly correlated with cell growth inhibition (Fig 2C inset)(R^2^ = 0.977; p < 0.001). Meanwhile, a reduction in spermidine was apparent only at the highest curcumin concentrations. As ODC inhibition typically results in the depletion of putrescine and spermidine pools with variable effects on spermine [26], the pattern observed in response to curcumin is inconsistent with ODC inhibition alone as the mechanism of polyamine depletion, suggesting an induction of polyamine catabolism as an additional regulatory component.

### Curcumin induces spermine catabolism through spermine oxidase

**Fig 3:**
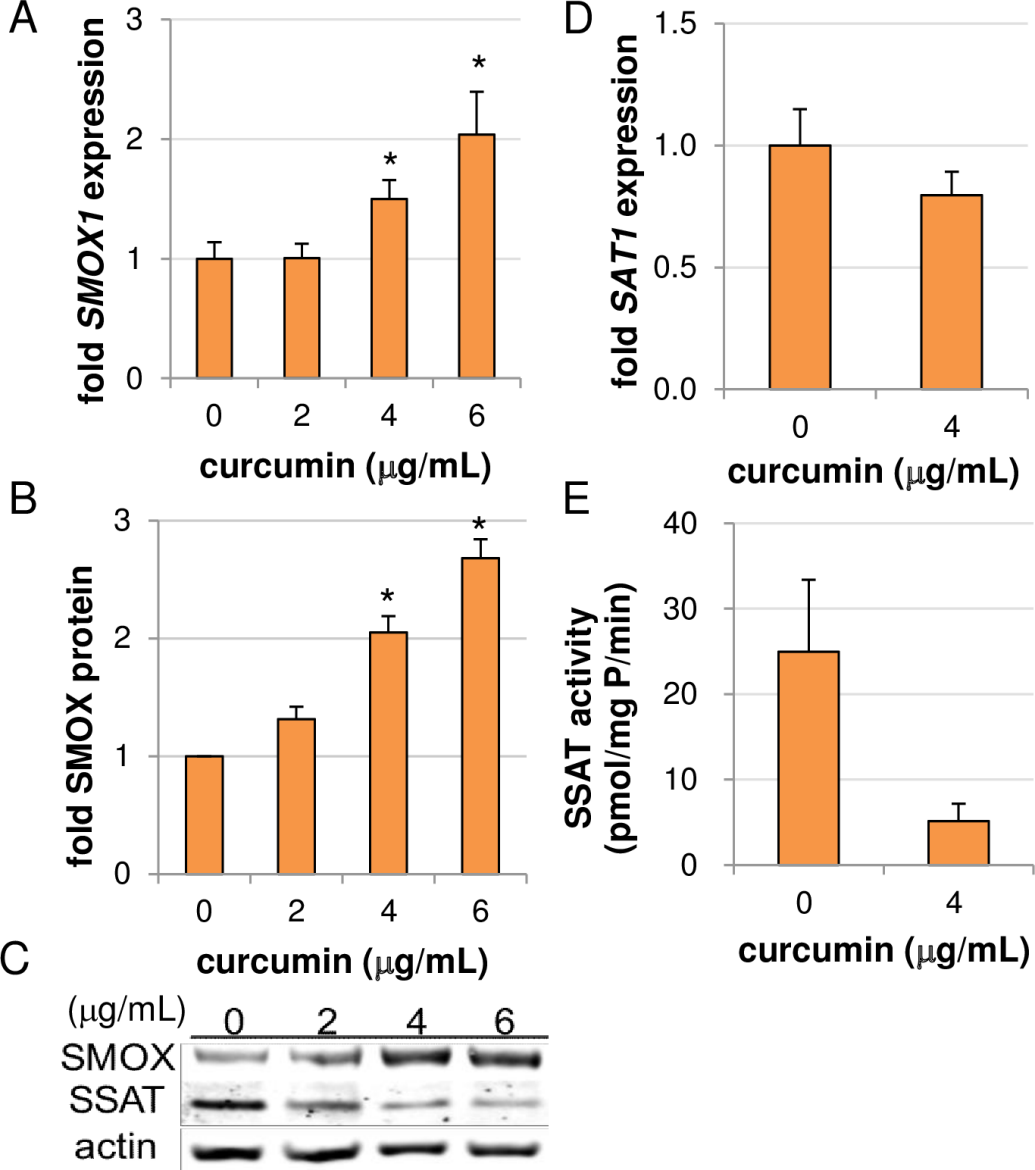
Curcumin induces spermine catabolism through spermine oxidation in AGS cells. AGS cells were treated for 48 hours and analyzed for SMOX (A) and SSAT (D) mRNA expression via qRT-PCR; (B-C) SMOX and SSAT protein levels by Western blot; and (E) SSAT enzyme activity. Graphs depict the mean values of at least 2 individual experiments, each measured in triplicate. Error bars = SEM; *p < 0.*05*. A representative western blot is presented in (C).

Due to this reduction in spermine, the effect of curcumin treatment on the polyamine catabolic pathway was investigated. Two options exist for the catabolism of spermine (Fig 1C): (1) acetylation by SSAT to form *N*^1^-acetylspermine, which is then either exported from the cell or oxidized back to spermidine via the peroxisomal N^1^-acetylpolyamine oxidase (PAOX); or (2) direct oxidation back to spermidine by spermine oxidase (SMOX), an enzyme localized both in the nucleus and cytoplasm that produces H_2_O_2_ and 3-aminopropanal as toxic byproducts. Of note, SMOX-generated H_2_O_2_ and its subsequent conversion into a reactive oxygen species (ROS) has been implicated in DNA damage and apoptosis of certain cancer cell types in response to antitumor polyamine analogues [27, 28]. As shown in Fig 3A–3C, treatment of AGS cells with curcumin up regulated both mRNA and protein levels of SMOX. In contrast, SSAT activity (Fig 3E) appeared to be down regulated post-transcriptionally, as curcumin had only a small, insignificant effect on SSAT mRNA abundance (Fig 3D). This is consistent with the loss of SSAT protein stabilization that occurs when polyamine pools are diminished [29]. The observed up regulation of SMOX correlates with the polyamine pool analysis (Fig 2C), suggesting that spermine oxidation contributes to the curcumin-mediated spermine depletion. Of note, curcumin interferes with numerous biochemical assays, including horseradish peroxidase (HRP)-based assays. As spermine oxidase assays measure the production of H_2_O_2_ using luminol- or homovanillic acid-based systems that incorporate HRP, the determination of SMOX enzyme activity in response to curcumin was not possible. Therefore, infrared-based quantitation of Western blots was used to measure SMOX protein induction (Fig 3B and 3C).

### Effects of curcumin on polyamine metabolism in HCT116 cells

**Fig 4:**
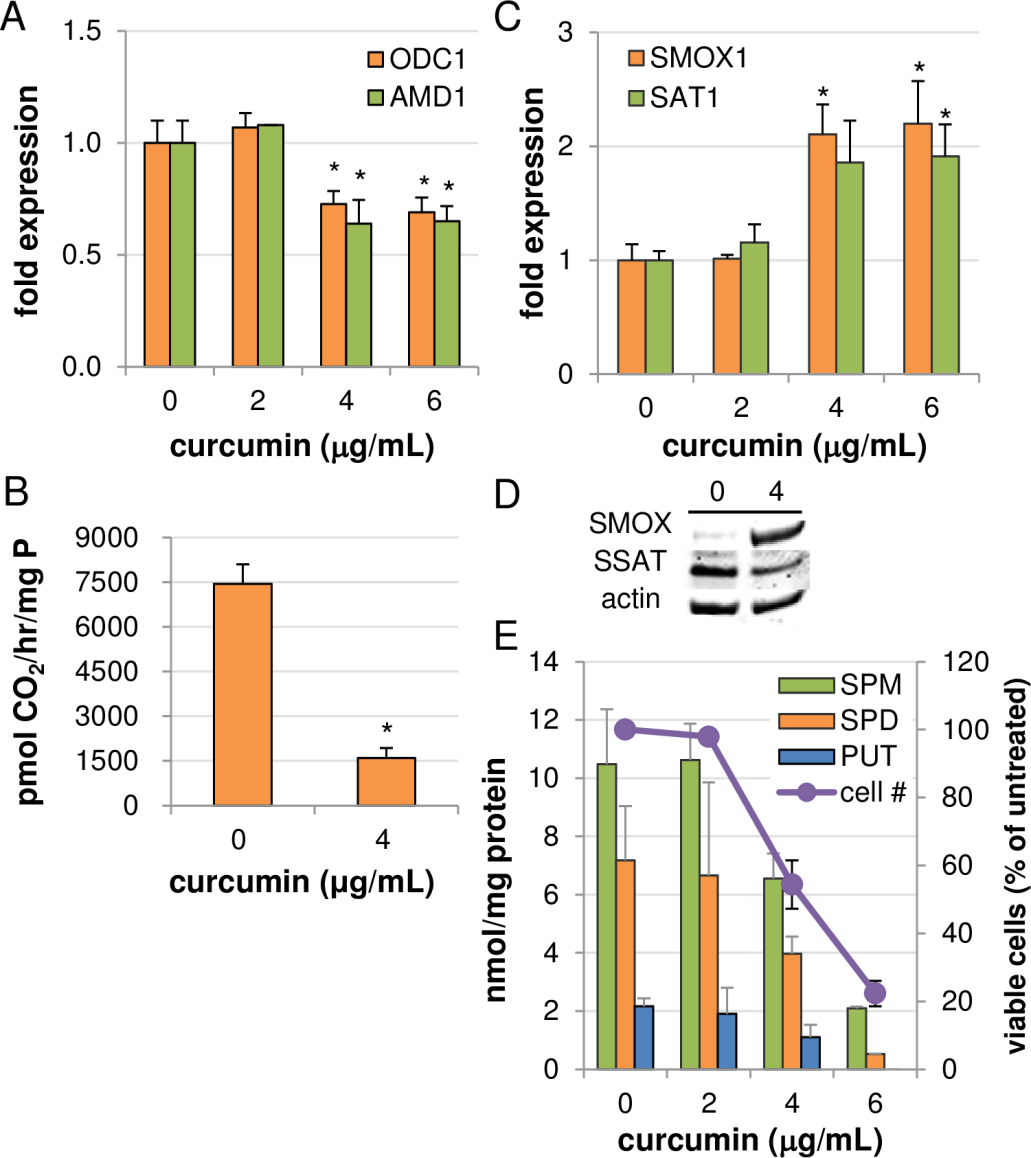
Effects of curcumin on polyamine metabolism in HCT116 colon cancer cells. RNA was collected from HCT116 cells treated with curcumin for 24 hours and used for qRT-PCR of genes encoding (A) the biosynthetic enzymes ODC and SAMDC (*AMD1*), or (C) the catabolic enzymes SMOX and SSAT (n = 3 biological experiments, each measured in triplicate). Total cell lysates were also collected and used for ODC enzyme activity assays (B; n = 2 independent experiments, each measured in triplicate) and Western blots of the catabolic enzymes (D). In (E), cells were treated for 48 hours with curcumin for either cellular proliferation assays or analysis of intracellular polyamine concentrations by HPLC (n ≥ 2, each measured in duplicate). All columns represent the means with error bars indicating SEM. *p < 0.*05* relative to untreated.

To further validate our findings in AGS cells, similar experiments were conducted in the HCT116 colon cancer cell line. Unlike AGS cells, HCT116 cells responded to curcumin treatment with decreases in mRNA levels of both ODC and SAMDC, suggesting transcriptional regulation by curcumin (Fig 4A). ODC enzyme activity following curcumin exposure was also decreased, with 4 μg/mL curcumin inhibiting ODC activity by approximately 80% over 24 hours (Fig 4B), substantially exceeding the transcriptional decrease observed and again suggesting an additional level of regulation. Additionally, SMOX mRNA expression and protein levels were induced, and although mRNA expression of SSAT was elevated, protein levels of SSAT decreased, as in AGS cells (Fig 4C and 4D). Analyses of individual polyamine pools following curcumin exposure revealed concurrent, dose-dependent reductions in putrescine, spermidine, and spermine, consistent with greater inhibition of the biosynthetic pathway than that observed in AGS cells as well as the enhancement of spermine catabolism. This reduction in polyamine pools was associated with inhibition of cell growth, as shown in Fig 4E.

### SMOX-generated H_2_O_2_ in the apoptotic response to curcumin

**Fig 5:**
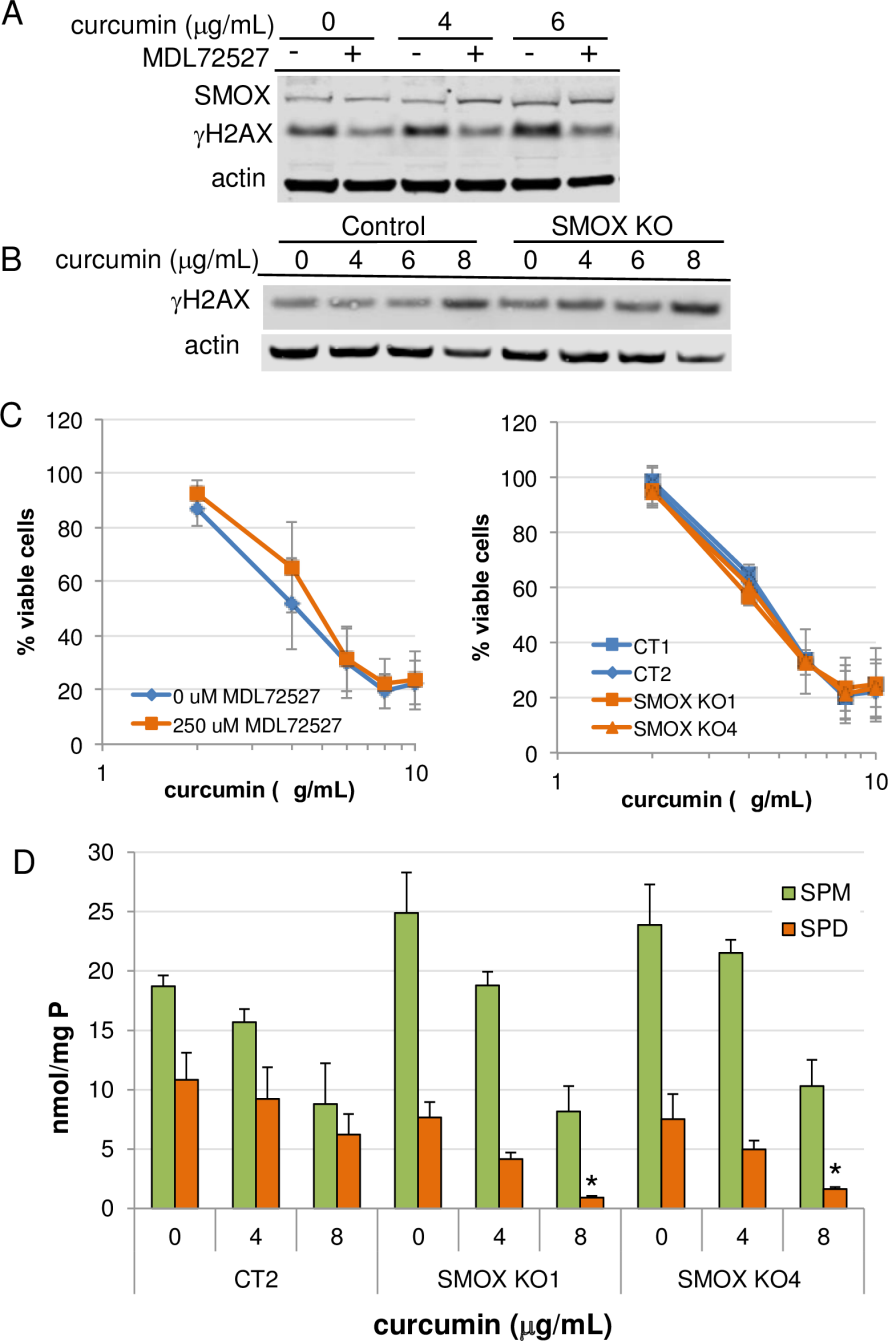
The effects of SMOX induction by curcumin on DNA damage and growth inhibition. (A) AGS cells were treated for 48 h with curcumin in the presence or absence of 250 μM MDL72527, a polyamine oxidase inhibitor. (B) SMOX knockout or control AGS cells were treated for 48 h with increasing concentrations of curcumin. Lysates were analyzed for changes in γH2AX by Western blot. (C) MTS assays were conducted with the same conditions as those in (A) and (B) to determine the effect of SMOX on curcumin-induced growth inhibition. (D) Intracellular spermine (SPM) and spermidine (SPD) levels of SMOX KO cells lines following curcumin exposure for 48 h. Data points indicate the means; error bars represent SEM; n = 3. CT1 and CT2 = CRISPR control AGS cell lines.

Curcumin induces apoptosis through the generation of reactive oxygen species. As a potential ROS, H_2_O_2_ is produced during spermine oxidation, which occurs in the nucleus in close proximity to DNA and chromatin [30], increasing the likelihood of oxidative DNA damage. We therefore investigated the contribution of curcumin-induced SMOX activity in the cytotoxic response of AGS cells using the polyamine oxidase inhibitor MDL72527 as well as CRISPR-generated genetic SMOX knockout lines. The accumulation of γH2AX, an indicator of DNA damage, was analyzed following curcumin exposure. Although treatment with MDL72527 decreased background levels of γH2AX, no effect was observed on that generated as a result of curcumin exposure (Fig 5A). Likewise, genetic knockout of SMOX in AGS cells failed to reduce the accumulation of DNA damage in response to curcumin (Fig 5B). Furthermore, MTS cell proliferation assays of either wildtype AGS cells cotreated with curcumin and MDL72527 or SMOX knockout clones treated with curcumin indicated no protection from its growth inhibitory effects (Fig 5C), indicating that ROS generated as a result of SMOX induction does not contribute significantly to the oxidative DNA damage or growth inhibition resulting from curcumin treatment. Intracellular polyamine pool analyses revealed reduced spermidine levels in the SMOX KO cell lines (Fig 5D). These results indicate a role for SMOX induction by curcumin as a source of spermidine and suggest that any benefit resulting from a reduction SMOX-generated ROS might be counteracted by increased loss of spermidine. Thus, it is likely that alternative mechanisms of ROS generation and growth inhibition are responsible for the DNA damaging and antiproliferative effects of curcumin.

### The role of reduced polyamines in the growth inhibitory response to curcumin

**Fig 6:**
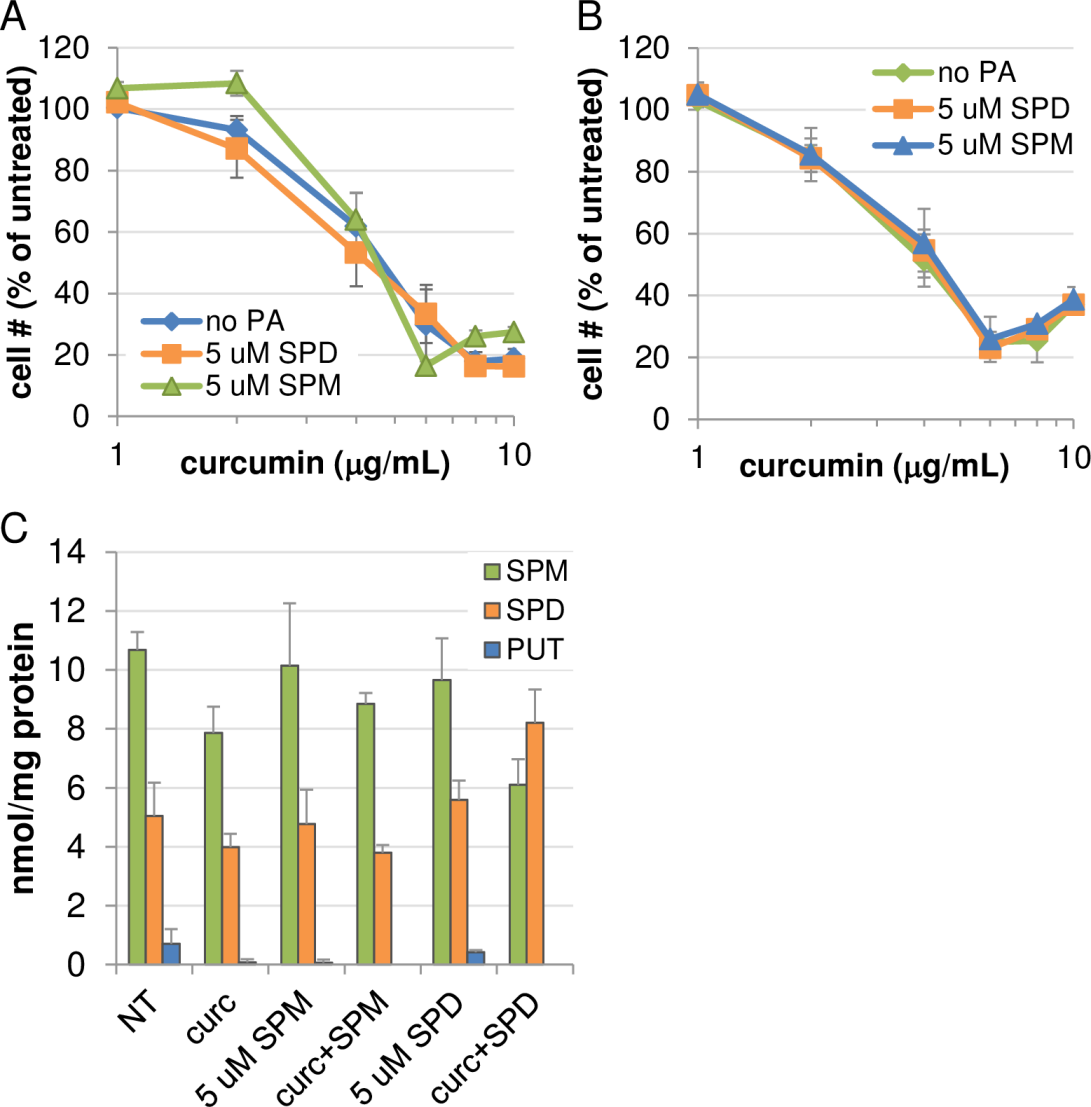
Polyamine rescue experiments. AGS (A) or HCT116 (B) cells were treated with curcumin for 48 hours in the presence or absence of spermidine or spermine and the bovine serum amine oxidase inhibitor aminoguanidine. MTS assays revealed that exogenous polyamines failed to protect cells from the growth inhibitory effects of curcumin. Data points indicate the means of at least 2 individual experiments, each measured in triplicate, with error bars representing SEM. (C) HPLC analysis of cotreated AGS cells confirmed uptake of spermine and spermidine into the cells, indicating that curcumin did not interfere with polyamine transport. Columns depict the means; error bars indicate SEM; n = 2, each measured in duplicate.

As mentioned, in both AGS and HCT116 cell lines, the reduction in intracellular polyamine contents closely reflected the inhibition of cell growth. Although reported by others that curcumin is capable of altering intracellular polyamine levels [15, 31], evidence has not been presented implicating or negating a role for polyamine depletion as a mechanism through which curcumin exerts growth inhibition. Therefore, rescue experiments were performed to determine if the addition of extracellular polyamines could reduce curcumin-mediated growth inhibitory effects. As shown in Fig 6, incubation of AGS (A) or HCT116 (B) cells for 48 hours in the presence of spermidine or spermine had no significant effect on proliferation in the presence of curcumin. To verify that polyamine transport was unaffected by curcumin exposure, AGS cells were treated concurrently with curcumin and either spermine or spermidine, and intracellular polyamines were analyzed by HPLC. Interestingly, the addition of spermidine into the culture medium in the presence of curcumin boosted the intracellular spermidine concentration and reduced the spermine concentration, relative to either curcumin or spermidine alone (Fig 6C). This result indicated that polyamine transport into the cell did occur in the presence of curcumin and suggested that, at least for spermidine, curcumin might enhance uptake. Spermine uptake at 48 h was less obvious, however, extending the cotreatment time to 72 h verified that spermine was also being transported and could normalize the curcumin-induced changes in polyamines (S1 Fig). Thus, curcumin-mediated growth inhibition appears to occur through mechanisms independent of polyamine depletion.

### Curcumin cooperates with ODC inhibition to reduce proliferation

**Fig 7:**
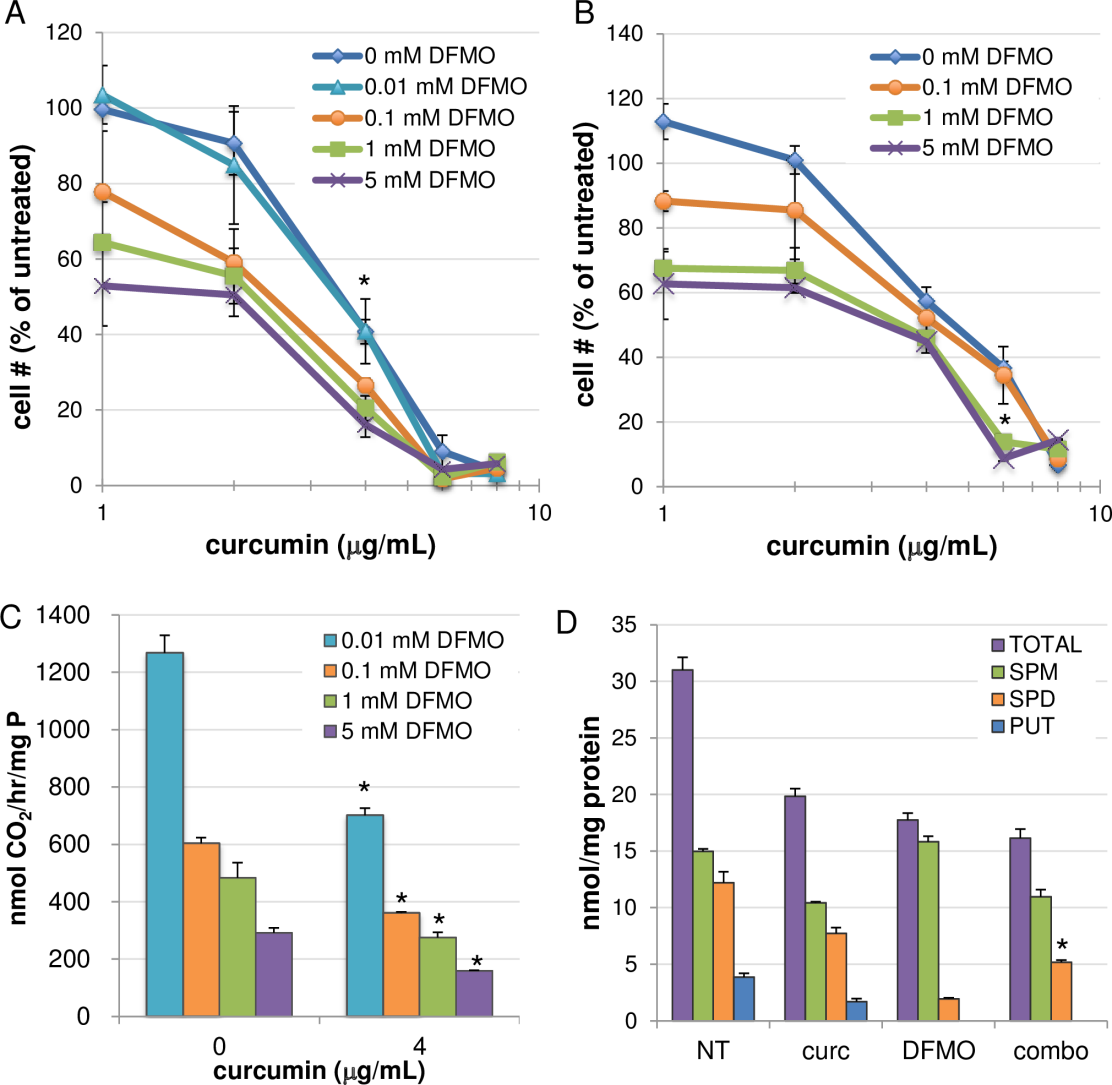
Curcumin cooperates with DFMO to inhibit cell growth. MTS cell proliferation assays revealed enhanced growth inhibition when combining curcumin with the ODC inhibitor DFMO in AGS (A) and HCT116 (B) cells. (C) In HCT116 cells, curcumin combined with DFMO for 24 hours enhanced ODC enzyme inhibition beyond that of single agents. Untreated and curcumin-treated ODC values (~7500 and 1600 pmol/hr/mg protein, respectively) are presented in Fig 4B. *p < 0.05 relative to DFMO alone; n = 2 individual experiments, each performed in triplicate; error bars = SEM. (D) Changes in individual and total polyamine pools following combination treatment of HCT116 cells for 24 h with 1 mM DFMO and 4 μg/mL curcumin. *p < 0.05 relative to SPD concentration following DFMO or curcumin alone. n = 2 individual experiments, each measured in duplicate; error bars = SEM. In (A), *p < 0.05 for combinations with 4 μg/mL curcumin and 1 or 5 mM DFMO versus the single agents. In (B), *p < 0.005 for the combination of 6 μg/mL curcumin and 1 mM DFMO, versus single agents. Data points indicate the means of at least 3 independent experiments; error bars = SEM.

Although the regulation of polyamine metabolism is indeed one of the multiple effects of curcumin in GI cancer cells, it does not appear to play a predominant mechanistic role in the antiproliferative or apoptotic response. However, the knowledge obtained in the studies described thus far provided rationale for exploiting polyamine metabolism as a potential drug target in combination with curcumin. DFMO, a suicide inhibitor of ODC, was examined in cell growth assays in combination with curcumin. As shown in Fig 7A, the addition of curcumin significantly increased the growth inhibitory effects of DFMO over 72 hours in AGS cells. Specifically, treatment with the combination of 1 mM DFMO and 4 μg/mL curcumin (the lowest concentration at which effects on polyamine metabolism were observed) inhibited cell growth by approximately 80%, an increase of ~40% over DFMO alone and approximately 20% beyond curcumin alone. Similarly, while 1 mM DFMO inhibited cell growth of HCT116 cells by approximately 27%, adding curcumin at 6 μg/mL inhibited cell growth by nearly 85% (Fig 7B). It should be noted that in most tumor cell types, DFMO treatment results in a cytostatic rather than cytotoxic response. Therefore, the possibility that curcumin exacerbates this response may have implications for the clinical use of DFMO, particularly with regard to lowering the effective dose of DFMO and thus minimizing unwanted side effects.

**Fig 8:**
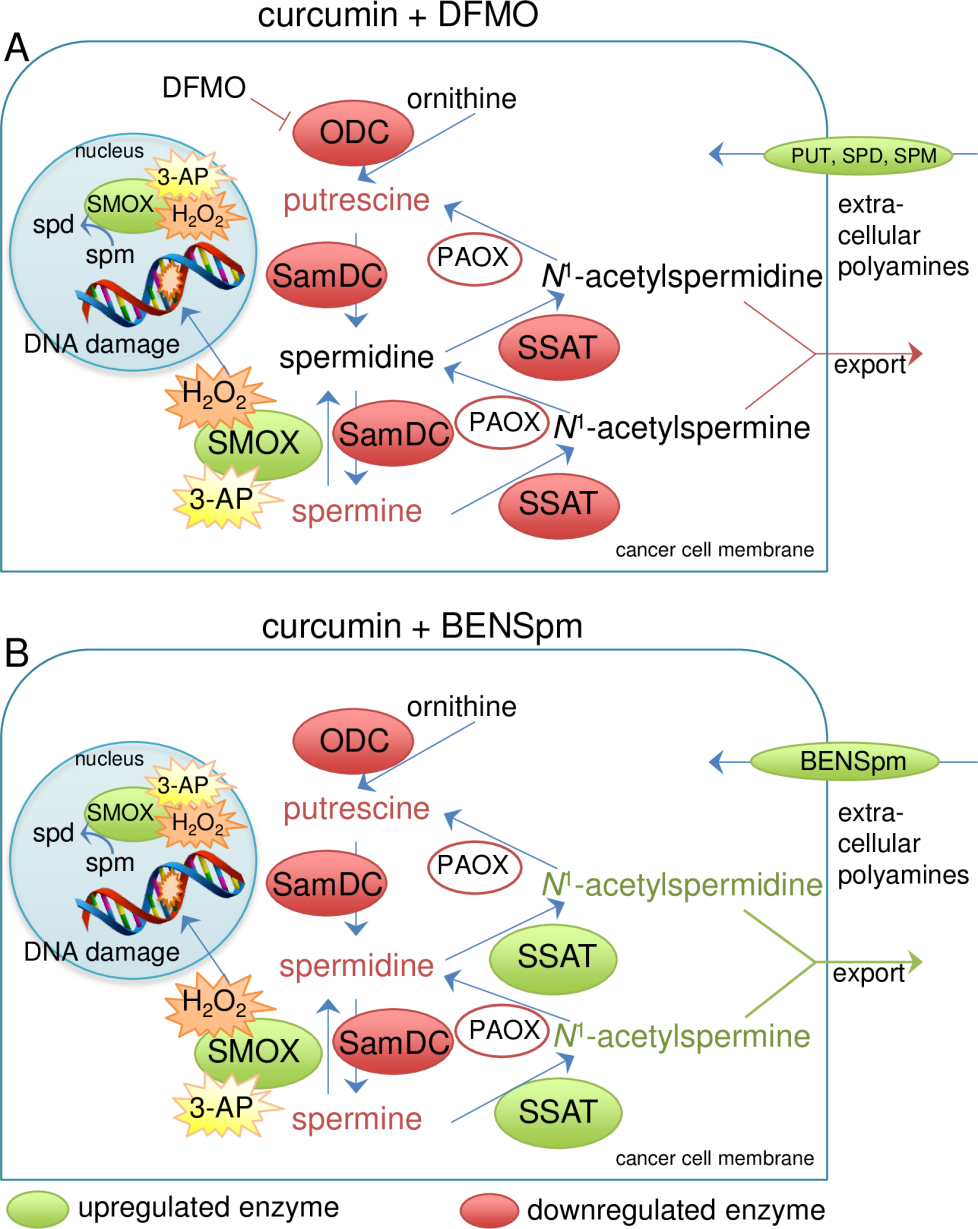
Proposed mechanisms of the combinatorial effects of curcumin with DFMO or BENSpm. Treatment with curcumin alone down regulates the biosynthetic enzymes ODC and SamDC, while up regulating SMOX. The result is decreased putrescine and the conversion of spermine back to spermidine. SSAT activity is lowered, likely due to the decrease in spermine. (A) Adding DFMO further inhibits ODC, reducing the spermidine generated through biosynthesis. Compensatory uptake of polyamines from the extracellular environment would likely be increased by both DFMO and curcumin. (B) Uptake of BENSpm through the polyamine transporter is stimulated by the curcumin-mediated decrease in biosynthesis. BENSpm induces catabolism through SSAT, thereby increasing the depletion of spermine and catabolizing the spermidine accumulated by the induction of SMOX by curcumin. As no accumulation of putrescine is evident, the acetylated polyamines are presumably exported from the cell.

As HCT116 cells maintain high basal levels of ODC activity, we used this cell line to further investigate the effects that combining curcumin with DFMO had on ODC enzyme activity and intracellular polyamine pools. After 24 h, cotreatment with curcumin and DFMO reduced ODC enzyme activity significantly beyond that of either agent alone (Fig 7C), resulting in a 98% inhibition of activity with DFMO at 5 mM. Notably, adding curcumin to each DFMO concentration resulted in ODC inhibition equal to that of an approximately 10-fold greater concentration of DFMO when used alone (e.g., 0.1 mM DFMO with curcumin provided a level of inhibition similar to that of 1 mM DFMO alone). The mean ODC activity level for untreated HCT116 cells in these experiments was approximately 7500 pmol CO_2_/mg protein/hour, while curcumin alone decreased ODC activity to ~1600 pmol CO_2_/mg protein/hour, as shown in Fig 4B. Polyamine levels in HCT116 cells following the 24-h combination treatment reflected the SMOX-mediated conversion of spermine to spermidine in combination with the DFMO- and curcumin-mediated inhibition of biosynthesis, resulting in an intermediate level of spermidine that was significantly less than that of curcumin treatment alone and greater than DFMO treatment (Fig 7D). The overall effects of the DFMO/curcumin combination treatment are illustrated in Fig 8A.

### Curcumin sensitizes cells to polyamine analogue treatment

**Fig 9:**
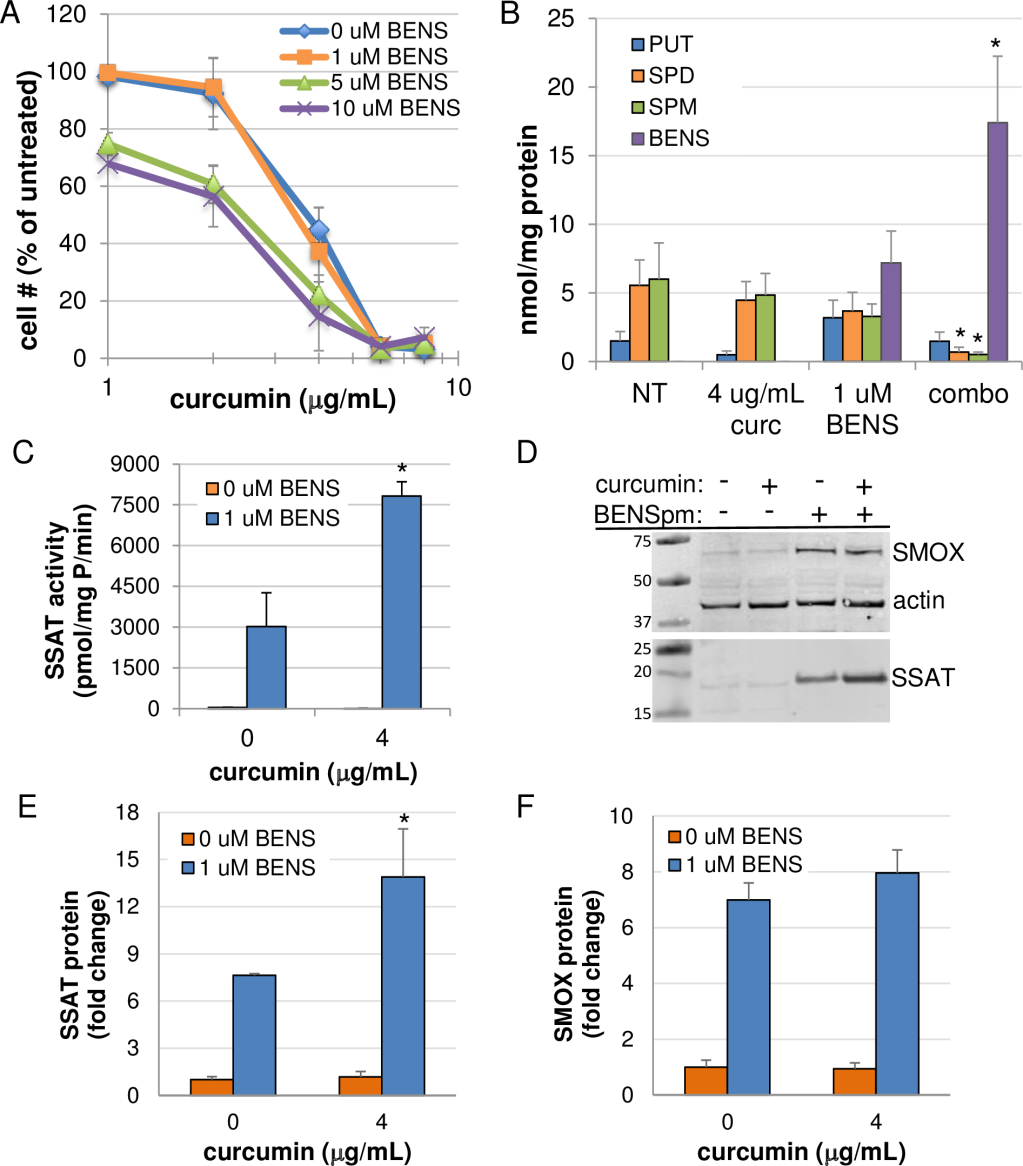
Curcumin cooperates with BENSpm to inhibit AGS cell growth. (A) MTS cell proliferation assays demonstrated enhanced growth inhibition over 72 h when combining curcumin with the polyamine analogue BENSpm in AGS cells. (B) HPLC analyses revealed increased accumulation of BENSpm in the presence of curcumin, resulting in significant decreases in spermidine and spermine pools. (C) SSAT activity is increased with curcumin/BENSpm cotreatment. (D) Representative Western blot indicating SMOX and SSAT protein levels following combination treatment. (E and F) Infrared imaging-based quantification of SSAT and SMOX Western blots, respectively, relative to actin. In A-F, data points indicate the means of at least 2 independent experiments, measured ≥ 2 times; error bars = SEM. *p < 0.05 relative to single agent treatments.

Members of the bis(ethyl) class of polyamine analogues use the polyamine transport system for cellular entry and are capable of reducing polyamine pools through the induction of polyamine catabolism combined with feedback inhibition of biosynthesis [32]. Cellular proliferation assays combining curcumin with the well-studied analogue BENSpm (Fig 1B) demonstrated reduced growth relative to either agent alone in both AGS and HCT116 cells (Fig 9A and S2A Fig, respectively). Analysis of intracellular polyamine pools revealed statistically significant increases in the accumulation of BENSpm in the presence of curcumin over the 72-hour treatment, and these increases coincided with decreases in spermine and spermidine that significantly exceeded those observed with either curcumin or BENSpm alone (Fig 9B and S2B Fig). These results are consistent with previous studies in which blocking polyamine biosynthesis with specific enzyme inhibitors, such as DFMO, caused increased uptake of polyamines from the extracellular environment [26, 33], but these are the first data indicating this ability for curcumin. As our results (Fig 2A and Fig 4B) and others indicate that curcumin inhibits ODC activity, it is likely that these cells up regulate polyamine transport in order to maintain optimal levels. However, by transporting BENSpm rather than a natural polyamine, polyamine catabolism is induced, further depleting the intracellular concentrations of spermine and spermidine. It is noteworthy that these studies were conducted using 1 μM BENSpm, a dose that, when used as a single agent has no effect on cell growth and elicits minor reductions in the natural polyamine pools. Although BENSpm stimulates both SSAT and SMOX activities, the reduction in both spermine and spermidine suggest a major role for increased SSAT induction as a result of increased analogue uptake in the presence of curcumin. Indeed, SSAT protein and activity were significantly increased when BENSpm was combined with curcumin treatment (Fig 9C–9E). The combination only slightly increased the abundance of SMOX protein beyond that of either agent alone (Fig 9D and 9F). Furthermore, proliferation assays using BENSpm and curcumin in the presence of either pharmacological SMOX inhibition or in SMOX KO cells indicated that induction of SMOX by the combination did not significantly contribute to its inhibitory effects (S3 Fig). The proposed overall mechanism of cooperation between curcumin and BENSpm is illustrated in Fig 8B.

## Discussion

Numerous flavonoids commonly found in the diet, including curcumin, can affect polyamine metabolism (reviewed in [14, 34]). Our finding that curcumin inhibits ODC and polyamine biosynthesis in cell lines of gastrointestinal cancer origin is consistent with those reporting ODC inhibition in breast cancer [15, 16] and leukemia cell lines [17]. Although not surprising, this inhibition alone is significant, as ODC activity is often used as a biomarker for tumor progression in animal models of carcinogenesis, and the ability of an agent to inhibit the induction of ODC in these models is associated with its chemopreventive potential [12]. Our novel finding that curcumin simultaneously induces spermine oxidation and reduces intracellular spermine pools, while inhibiting biosynthesis, distinguishes its action from that of the commonly used ODC inhibitor DFMO, which often depletes only putrescine and spermidine. The implications of this could be several, including an increase in oxidative stress, as spermine is a known free radical scavenger [35] and hydrogen peroxide is produced during spermine oxidation [36].

Aside from effects on polyamine metabolism, curcumin shares important anti-inflammatory characteristics with non-steroidal anti-inflammatory drugs (NSAIDs), such as aspirin and sulindac, including the ability to inhibit cyclooxygenase-2 (COX2), a defining characteristic of traditional NSAIDs [37]. Key to its anti-inflammatory functions, curcumin inhibits activation of the transcription factor nuclear factor kappa B (NF-κB) [38, 39], which targets promoter regions of inflammation- and proliferation-associated genes. NF-κB-regulated gene products include enzymes that produce free radicals, such as inducible nitric oxide synthase (iNOS), lipoxygenase (LOX), and COX2, pro-inflammatory cytokines, such as tumor necrosis factor alpha (TNFα) and interleukins (IL) 1 and 6, and growth-promoting factors such as c-MYC and cyclin D1. As the genes encoding ODC and SAMDC are both driven by c-MYC [40, 41], it is possible that curcumin-mediated inhibition of c-MYC activation might play a role in the transcriptional down regulation of these genes observed in HCT116 cells, which possess high levels of c-MYC. Studies have also shown ODC and SSAT to be regulated by NF-κB [42, 43], although the loss of SSAT activity observed in our cells with curcumin treatment appeared to be due to a post-transcriptional mechanism. Conversely, the high levels of polyamines in tumor cells have been associated with the activation of NF-κB nuclear translocation [44]. Therefore, the reduction of intracellular polyamines by curcumin could hypothetically mediate its inhibition of NF-κB activation. This ability of curcumin to mediate key factors linking inflammation and cancer is a likely basis for many of its anticarcinogenic activities. Thus, *in vivo*, in addition to inhibiting polyamine biosynthesis, curcumin may provide added benefits similar to those of NSAIDs through its anti-inflammatory capabilities.

Our current data demonstrating enhanced ODC inhibition and growth arrest when adding curcumin to DFMO indicate the potential of this combination as a chemopreventive regimen. ODC and polyamine levels in neoplastic or tumor tissue are generally increased compared to adjacent normal tissue. In particular, polyps of the colon have elevated polyamine biosynthesis relative to normal colonic mucosa [45–48]. The progression of adenoma to carcinoma is associated with mutation of the *adenomatous polyposis coli (APC)* gene, which has been detected in 70–80% of sporadic colorectal carcinoma patients and is the major player in several hereditary colon cancer syndromes [49, 50]. Importantly, this mutation results in simultaneous up regulation of ODC as well as COX-1 and COX-2, the therapeutic targets of DFMO and most NSAIDs, respectively, which are also inhibited by curcumin. In a mouse model of familial adenomatous polyposis (FAP) resulting from a mutated *Apc* allele, dietary curcumin intake decreased adenoma formation by 64% [51]. Dietary curcumin also prevented azoxymethane (AOM)-induced ODC activity in the colonic mucosa of rats, and this was associated with a decreased incidence (> 50%) of early preneoplastic lesions in the form of aberrant crypt foci [52]. This same study also reported reductions in AOM-induced LOX and COX metabolites, including prostaglandin E_2_, in the colonic mucosa of rats receiving dietary curcumin, suggesting that the effects of curcumin overlap with those of NSAIDs in preventing inflammation-associated tumorigenesis. Therefore, curcumin has the potential to limit the activities of key enzymes in individual pathways known to promote carcinogenesis.

The value of our data implicating an enhanced antitumor response when supplementing DFMO treatment with curcumin indicate the potential of this combination as a chemopreventive regimen that would allow reduction of DFMO dose and avoidance of side effects associated with NSAID usage. As chemoprevention involves the long-term administration of a natural or synthetic compound to healthy individuals with the goal of preventing or delaying the onset of disease, the minimization of potential side effects is critical. The overall safety of DFMO has been well established, and it currently retains orphan drug FDA status with approval for the treatment of human African trypanosomiasis and female hirsutism [53]. Although its long-term use was initially associated with a dose-related, reversible ototoxicity, anemia, and mild gastrointestinal side effects, dose de-escalation studies focusing on prevention rather than cancer treatment revealed that DFMO dosage could be reduced to that which minimizes these off-target effects while effectively reducing colorectal mucosa polyamine levels in patients susceptible to colorectal cancer [13, 54]. Notably, in a randomized, placebo-controlled Phase III clinical trial, oral administration of DFMO along with the NSAID sulindac dramatically reduced the occurrence of colorectal adenomas in susceptible patients [55]. In addition to their anti-inflammatory effects, NSAIDs also induce polyamine catabolism through SSAT activity; therefore, combining them with DFMO depleted the levels of intracellular polyamines beyond those required for aberrant proliferation [56], while also providing anti-inflammatory benefits. These combination treatments with NSAIDs have generally allowed a lowering of the effective dose of DFMO required. However, long-term NSAID use can result in injury to the gastrointestinal mucosa as well as renal toxicity, with less frequent but serious side effects manifesting in the cardiovascular and central nervous systems [57]. By cooperating with DFMO to reduce intracellular polyamine concentrations, including spermine, while exerting its anti-inflammatory effects and other modes of action independent of polyamine depletion, curcumin might serve as a safe and perhaps more effective substitute for NSAIDs in the chemopreventive setting, particularly for individuals at greater risk for side effects.

Cancer cells, unlike non-tumorigenic cells, generally respond to curcumin exposure with apoptotic cell death [3]. The mechanisms underlying this cancer-cell selectivity include increased curcumin uptake, increased generation of reactive oxygen species (ROS) coincident with reduced levels of intracellular glutathione, and interference with the cancer cell’s reliance on survival signals resulting from constitutive activation of NF-κB [3, 58–60]. In our cancer cell line studies, combining curcumin with DFMO exaggerated the cytostatic response of DFMO into a more cytotoxic one, and we also demonstrated that curcumin has additional modes of action that are independent of polyamine depletion. Furthermore, our data also suggest utility for curcumin/polyamine analogue combinations in the chemotherapeutic arena (Fig 8B). The bis(ethyl) class of polyamine analogues, including bis(ethyl)norspermine (BENSpm, Fig 1B), serve as structural mimetics that compete with the natural polyamines for uptake and binding within the cell. Their accumulation results in a dramatic up regulation of polyamine catabolism while downregulating biosynthesis, depleting the cell of its natural polyamine pools. Importantly, these analogues are unable to fulfill the functional requirements of the natural polyamines, resulting in apoptosis and cell death. As shown in Fig 9 and illustrated in Fig 8B, exposure to curcumin lowered the dose of the bis(ethyl) polyamine analogue BENSpm required to deplete polyamines and arrest growth. This effect was correlated with increased accumulation of BENSpm that was likely due to compensatory uptake in response to the down regulation of biosynthesis by curcumin. Interestingly, this combined reduction of polyamine biosynthesis by curcumin with induction of catabolism and polyamine depletion may have similar effects to those of combining DFMO with NSAIDs, as NSAIDs also induce SSAT to further reduce polyamine pools [56]. The clinical utility of BENSpm has been hampered by dose-limiting toxicities [61]; however, the ability of curcumin to enhance its uptake and accumulation in tumor cells may allow for a reduction in the dosage required for polyamine depletion. Furthermore, a second-generation bis(ethyl) polyamine analogue, PG-11047, has been developed to minimize off-target effects while retaining the functional abilities of BENSpm [62]. PG-11047 has a significantly improved safety profile and is well tolerated in clinical trials (clinicaltrials.gov).

The safe consumption of curcumin is evident in its centuries-long use in traditional medicines as well as through its investigation in nearly 70 completed clinical trials to date (clinicaltrials.gov). In spite of an abundance of promising preclinical studies in many fields of medicine, the clinical success of curcumin in regulated trials has been lacking. Of particular relevance, a double-blind, placebo-controlled clinical trial was recently completed investigating the effect of dietary curcumin administration on the regression of adenomatous polyps in FAP patients [63]. After 1 year, there were no significant differences in polyp number or size between the curcumin and placebo groups. Likewise, polyamine levels in the colonic mucosa of curcumin-treated patients did not significantly differ from those patients receiving placebo. These results are likely due to the known pharmacological limitations of curcumin, including poor solubility and stability and the low overall bioavailability of pure curcumin [2, 64]. Although direct contact with the GI tract potentially lessons the requirement for systemic bioavailability, the results of the abovementioned study still indicate a need for improved pharmacokinetics. Fortunately, several promising strategies are being investigated, including the use of adjuvants that impede curcumin metabolism, curcumin-containing nanoparticles, and structural analogues of curcumin [2, 64].

In conclusion, we have demonstrated that curcumin mediates intracellular polyamine levels in gastrointestinal cancer cell lines through down regulating biosynthesis while up regulating spermine oxidation. Curcumin cooperates with DFMO, an ODC inhibitor approved for clinical use, to further decrease polyamine biosynthesis and inhibit cell growth, suggesting the potential for curcumin to substitute for NSAIDs in a chemopreventive regimen. Furthermore, through down regulating biosynthesis, curcumin can stimulate uptake of antitumor polyamine analogues, resulting in lower doses required to deplete polyamines and induce tumor cell death. Thus, combining curcumin with modulators of polyamine metabolism holds promise as novel strategies in both chemoprevention and chemotherapeutics.

## Supporting information

S1 Fig Exogenous spermine replenishes intracellular pools. AGS (A) and HCT116 (B) cells were treated with curcumin for 72 hours in the presence or absence of 5 μM spermine and the bovine serum amine oxidase inhibitor aminoguanidine. (B) HPLC analyses confirmed that spermine levels were restored, indicating that curcumin did not interfere with polyamine transport. Columns represent the means of 2 replicates; error bars indicate standard deviations.
(TIF)

S2 Fig Curcumin cooperates with BENSpm to inhibit HCT116 cell growth. (A) MTS cell proliferation assays demonstrated enhanced growth inhibition over 72 h when combining curcumin with the polyamine analogue BENSpm in HCT116 cells. (B) HPLC analyses revealed increased accumulation of BENSpm in the presence of curcumin, resulting in significant decreases in spermidine and spermine pools. Data points indicate the means of at least 2 independent experiments, measured ≥ 2 times; error bars = SEM. *p < 0.05.
(TIF)

S3 Fig The combined growth inhibitory effect of BENSpm and curcumin is independent of SMOX activity. AGS cells were treated with curcumin in the presence or absence of pharmacologic (A) or genetic (B) SMOX inhibition and analyzed for growth inhibition by MTS assays. Data points indicate means; error bars represent SD.
(TIF)
