# Supplementary figures and images for: Curcumin mediates polyamine metabolism and sensitizes gastrointestinal cancer cells to antitumor polyamine-targeted therapies
Source: PLoS One. 2018 Aug 23;13(8):e0202677. doi: 10.1371/journal.pone.0202677 (PMC6107220; doi:10.1371/journal.pone.0202677)

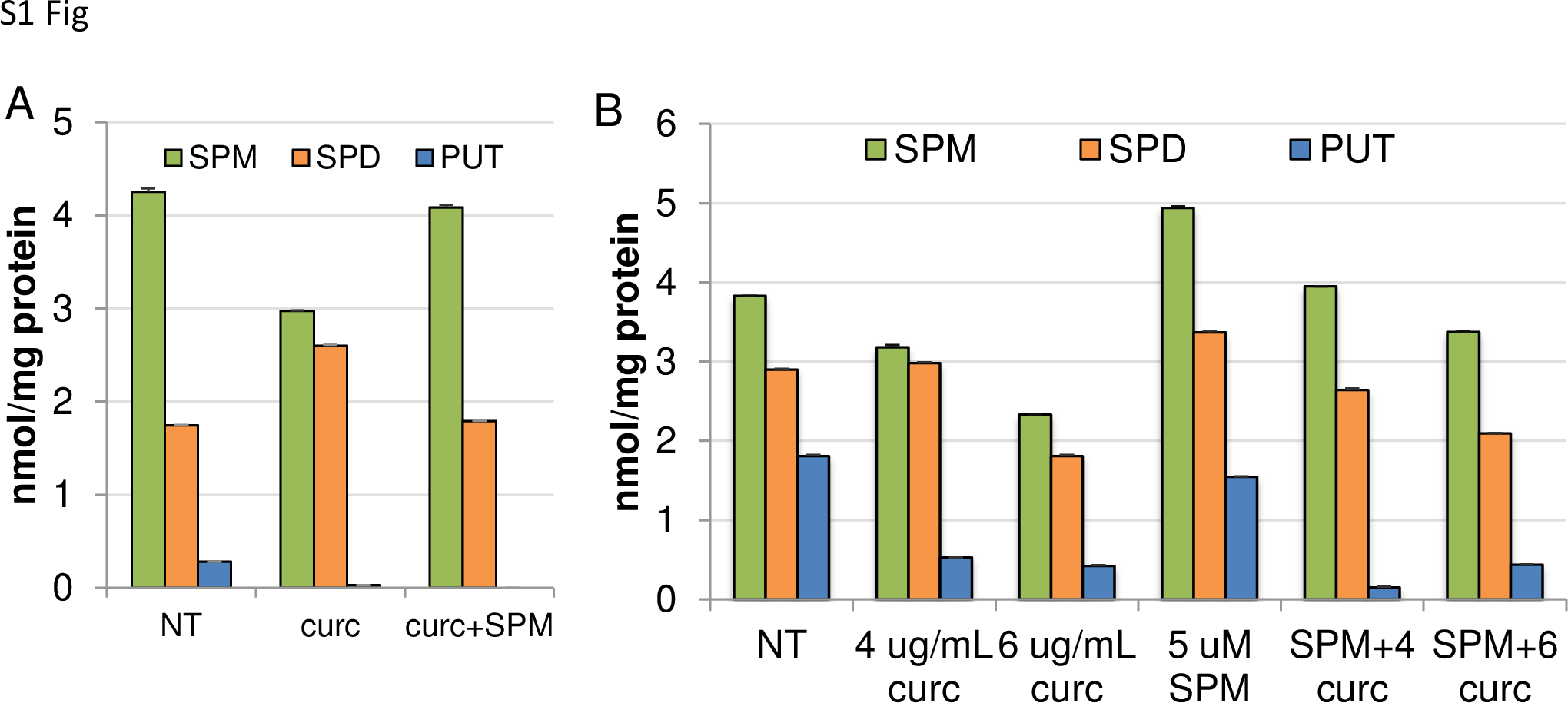

Supplement: S1 Fig — AGS (A) and HCT116 (B) cells were treated with curcumin for 72 hours in the presence or absence of 5 μM spermine and the bovine serum amine oxidase inhibitor aminoguanidine. (B) HPLC analyses confirmed that spermine levels were restored, indicating that curcumin did not interfere with polyamine transport. Columns represent the means of 2 replicates; error bars indicate standard deviations. (TIF) [file pone.0202677.s001.tif]

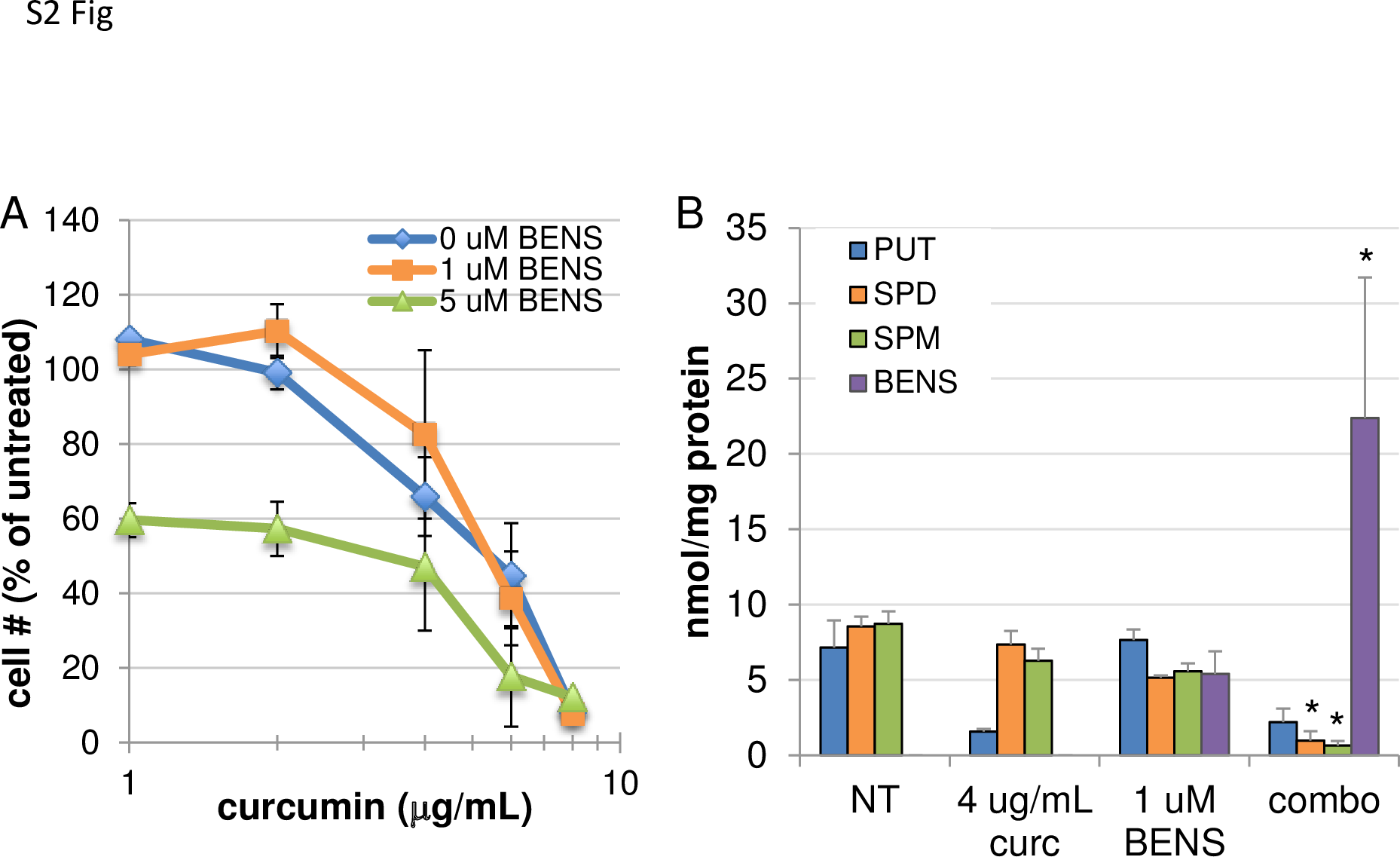

Supplement: S2 Fig — (A) MTS cell proliferation assays demonstrated enhanced growth inhibition over 72 h when combining curcumin with the polyamine analogue BENSpm in HCT116 cells. (B) HPLC analyses revealed increased accumulation of BENSpm in the presence of curcumin, resulting in significant decreases in spermidine and spermine pools. Data points indicate the means of at least 2 independent experiments, measured ≥ 2 times; error bars = SEM. *p < 0.05. (TIF) [file pone.0202677.s002.tif]

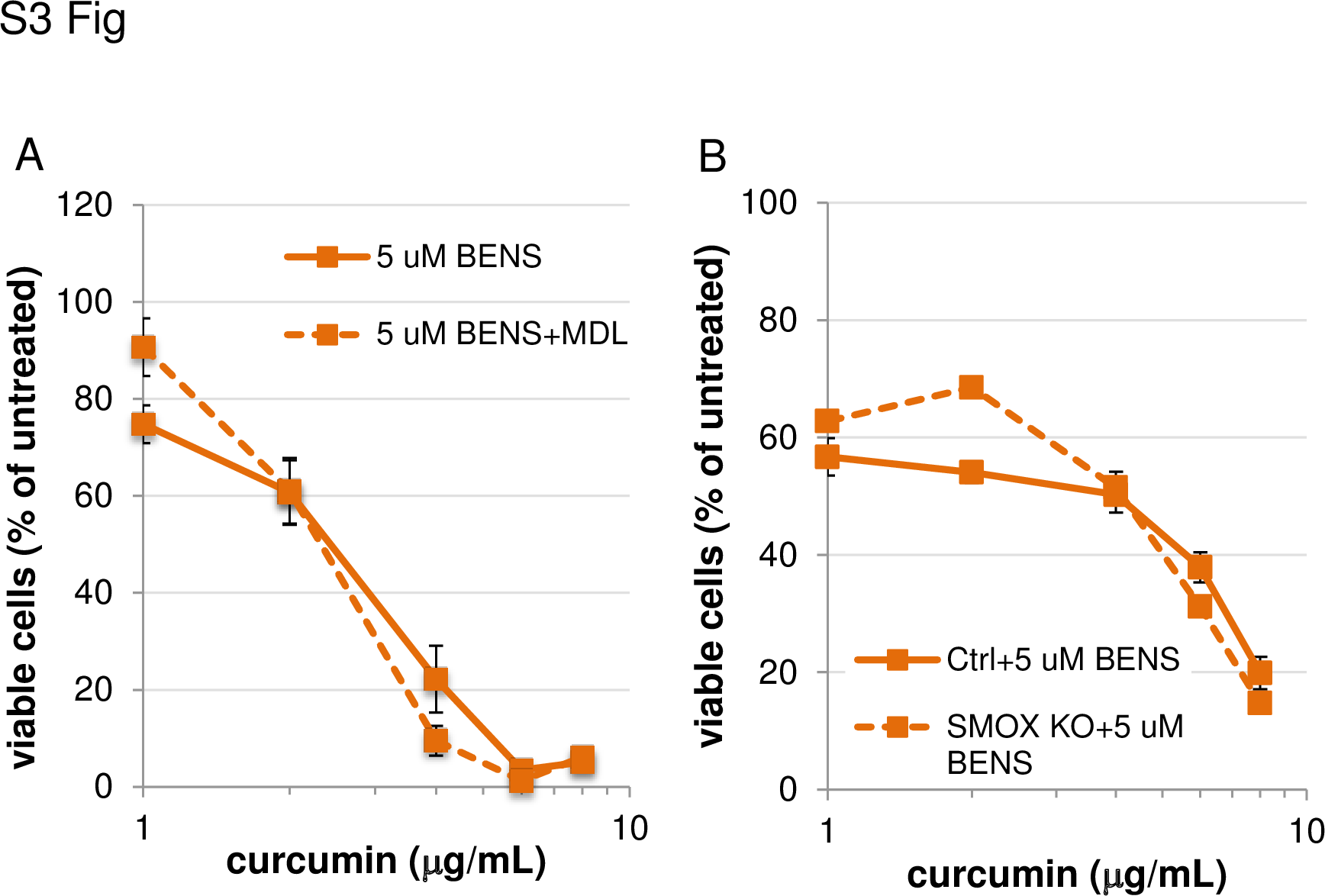

Supplement: S3 Fig — AGS cells were treated with curcumin in the presence or absence of pharmacologic (A) or genetic (B) SMOX inhibition and analyzed for growth inhibition by MTS assays. Data points indicate means; error bars represent SD. (TIF) [file pone.0202677.s003.tif]
